# Serotonin modulates insect gut bacterial community homeostasis

**DOI:** 10.1186/s12915-022-01319-x

**Published:** 2022-05-13

**Authors:** Tian Zeng, Hong-ai Su, Ya-lan Liu, Jian-fang Li, Ding-xin Jiang, Yong-yue Lu, Yi-xiang Qi

**Affiliations:** 1 Department of Entomology, College of Plant Protection, South China Agricultural University, Wushan Road 483, Tianhe District, Guangzhou, 510642 China; 2 Key Laboratory of Natural Pesticide and Chemical Biology, Ministry of Education, Laboratory of Insect Toxicology, South China Agricultural University, Guangzhou, China

**Keywords:** Serotonin, Phenylalanine hydroxylase, Dual oxidase, Gut microbiome, Homeostasis, *Serratia*, *Providencia*

## Abstract

**Background:**

Metazoan guts are in permanent contact with microbial communities. However, the host mechanisms that have developed to manage the dynamic changes of these microorganisms and maintain homeostasis remain largely unknown.

**Results:**

Serotonin (5-hydroxytryptamine [5-HT]) was found to modulate gut microbiome homeostasis via regulation of a dual oxidase (*Duox*) gene expression in both *Bactrocera dorsalis* and *Aedes aegypti*. The knockdown of the peripheral 5-HT biosynthetic gene phenylalanine hydroxylase (*TPH*) increased the expression of *Duox* and the activity of reactive oxygen species, leading to a decrease in the gut microbiome load. Moreover, the *TPH* knockdown reduced the relative abundance of the bacterial genera *Serratia* and *Providencia*, including the opportunistic pathogens, *S. marcescens* and *P. alcalifaciens* in *B. dorsalis*. Treatment with 5-hydroxytryptophan, a precursor of 5-HT synthesis, fully rescued the *TPH* knockdown-induced phenotype.

**Conclusions:**

The findings reveal the important contribution of 5-HT in regulating gut homeostasis, providing new insights into gut–microbe interactions in metazoans.

**Supplementary Information:**

The online version contains supplementary material available at 10.1186/s12915-022-01319-x.

## Background

The monoamine serotonin (5-hydroxytryptamine [5-HT]) is a neurotransmitter in the animal brain. It regulates a wide variety of processes in most invertebrates and vertebrates, such as feeding [1], aggression [2], learning and memory [3] in *Drosophila*; mood, behavior, sleep cycles, and appetite in humans [4]. Serotonin is also an important regulatory factor outside of the central nervous system in immune signaling [5]. In mammals, more than 90% of the 5-HT is biosynthesized in the gut, and the gut-derived 5-HT regulates diverse functions [6]. For example, 5-HT interacts with microbiota in the mammalian gut. Elevated levels of intestinal lumenal 5-HT increase the relative abundance of spore-forming members of the gut microbiota [7], which in turn can promote host 5-HT biosynthesis [8, 9]. However, the precise mechanisms by which 5-HT modulates gut microbiome remains to be determined.

The metazoan intestine harbors numerous microbial communities that influence multiple aspects of host physiology, such as immunity, nutrition, and development [10, 11]. The quantity and composition of gut microbial communities may fluctuate dynamically in response to host diet, physical activities, and environmental conditions [12, 13]. Therefore, gut epithelia must tolerate a certain amount of proliferation of commensal microbes in order for beneficial gut–microbe interactions to occur. In addition, the gut needs to evade many types of noncommensal microorganisms, including foodborne microorganisms and pathogens. To maintain the intestinal microbiome homeostasis, finely tuned immune systems have evolved in metazoan gut. For example, *Drosophila* gut immune systems are primarily dual oxidase (Duox)-dependent reactive oxygen species (ROS) production [14, 15] and an immune deficiency (IMD) pathway [16].

Dipteran insects, such as *Drosophila*, share common aspects of gut–microbe interactions and gut physiology with many other metazoans [17]. Because of the relative simplicity of the insect intestinal system, examining the interactions between serotonin and gut microbiome homeostasis may be easier in insects. In recent years, insect gut–microbe interactions have attracted increasing attention. Gut symbionts mediate insecticide resistance in the agricultural pest *Bactrocera dorsalis* [18], and disease transmission in vector mosquitoes [19, 20]. In this study, we conduct a comprehensive study in *B. dorsalis* and *Aedes aegypti* to elucidate the function of 5-HT in gut microbiome homeostasis. The blockage of peripheral 5-HT biosynthesis decreased the gut bacterial load and reduced the proportion of the bacterial genus *Serratia* in the gut of both the oriental fruit fly *B. dorsalis* and the vector mosquito *A. aegypti*. In addition, 5-HT regulated commensal bacterial proliferation through regulation of *Duox* expression.

## Results

### Effect of pharmacological elevation of serotonin on the gut microbiome in B. dorsalis

**Fig. 1:**
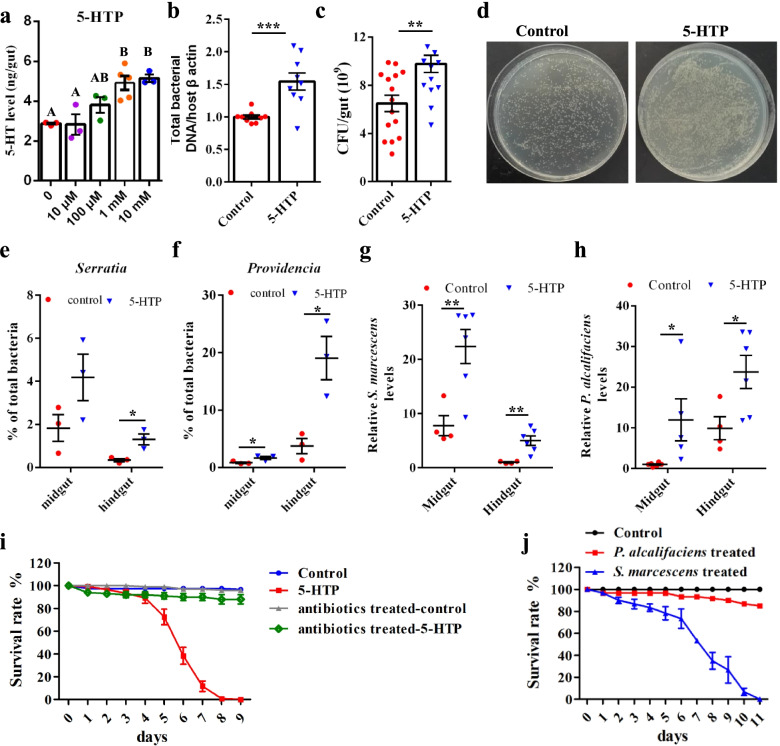
Effects of pharmacological elevation of serotonin on gut microbiome homeostasis and survival of *B. dorsalis*. **a** Dose–response curve for 5-HTP (4-day treatment). The error bar represents the standard error, and different letters above each bar indicate significant differences (*p* < 0.05) according to one-way ANOVA followed by Tukey’s multiple comparison test. **b**–**d** 5-HTP treatment increased the gut bacterial load, as determined by **b** 16S rRNA quantification using real-time PCR and **c**, **d** colony-forming unit assay. **e**, **f** Relative abundance of the genus *Serratia* and *Providencia* in control and 5-HTP-treated *B. dorsalis* by 16S rDNA sequencing. **g** 5-HTP treatment increased the load of *S. marcescens* and **h** *P. alcalifaciens* in the gut of *B. dorsalis*. The housekeeping *β-actin* gene was used as an endogenous control. **i** Survival of 5-HTP-treated *B. dorsalis* and controls. **j** Survival of *B. dorsalis* fed *S. marcescens* or *P. alcalifaciens*. Bacteria were introduced into fly gut via sugar meals. Flies fed with a diet supplemented with sterile 5% sucrose only served as a control. Surviving flies were counted daily. Two-tailed unpaired *t*-test was performed in b, c, e, f, g, and h. Error bars indicate ± s.e.m.; *p < 0.05, **p < 0.01

To test for a direct effect of 5-HT on the gut microbiome of the oriental fruit fly *B. dorsalis*, 5-HT levels were pharmacologically elevated by feeding flies 5-hydroxytryptophan (5-HTP), a precursor of 5-HT biosynthesis (Additional file 1: Fig. S1). First, a dose–response curve was generated for 5-HT levels in the intestinal tracts of flies that were fed different concentrations of 5-HTP for 4 days. The maximum effects occurred at 1 mM, and in flies treated with 1-mM 5-HTP, 5-HT increased approximately twofold over baseline levels (Fig. 1a).

Then, the effect of the elevated level of 5-HT on the gut microbiome was evaluated. The gut bacterial population was investigated using both a culture-independent method (16S rRNA quantification by real-time PCR) and colony-forming unit counts (cultivable bacteria). With the 5-HTP treatment, the microbiome load increased by approximately twofold (Fig. 1b). The cultivable bacteria in 5-HTP-treated flies also increased significantly, compared with those in the control flies (Fig. 1c, d).

Whether the increase in serotonin changed the composition of the gut microbial community in *B. dorsalis* was also determined. The midguts and hindguts were isolated from flies for bacterial 16S rDNA sequencing. Treatment with 5-HTP affected the gut bacteria composition at the genus level (Additional file 1: Fig. S2). We focus specifically on *Serratia* and *Providencia*, because species of these two genera are insect opportunistic pathogens [21, 22]. Intriguingly, the abundance of *Serratia* and *Providencia*, which mainly inhabited the midgut and hindgut, respectively, elevated after 5-HTP treatment (Fig. 1e, f). The relative abundance of the opportunistic pathogens *Serratia marcescens* (Otu000010) and *Providencia alcalifaciens* (Otu000007) increased significantly in 5-HTP-treated flies (Additional file 1: Fig. S3a–c). The increased burdens of *S. marcescens* and *P. alcalifaciens* were confirmed by RT-qPCR (Fig. 1g, h).

Because of the increase in load of opportunistic pathogens in the fly gut, the effect of the 5-HTP treatment on fly survival was tested. With the oral introduction of 5-HTP, fly mortality was 100% within 9 days (Fig. 1i). While the mortality rate of the axenic flies in the presence of 5-HTP ingestion was about 10% (Fig. 1i). Then, flies were treated with the *S. marcescens* and *P. alcalifaciens* isolated from the gut of *B. dorsalis*. In the flies that ingested *S. marcescens*, 100% mortality occurred within 11 days (Fig. 1j). By contrast, the survival rate of flies fed *P. alcalifaciens* was 85% over 11 days (Fig. 1j). These results indicated that the 5-HTP-induced disturbance of gut microbiota decreased host survival.

### Serotonin contributes to the maintenance of gut microbiota in B. dorsalis

**Fig. 2:**
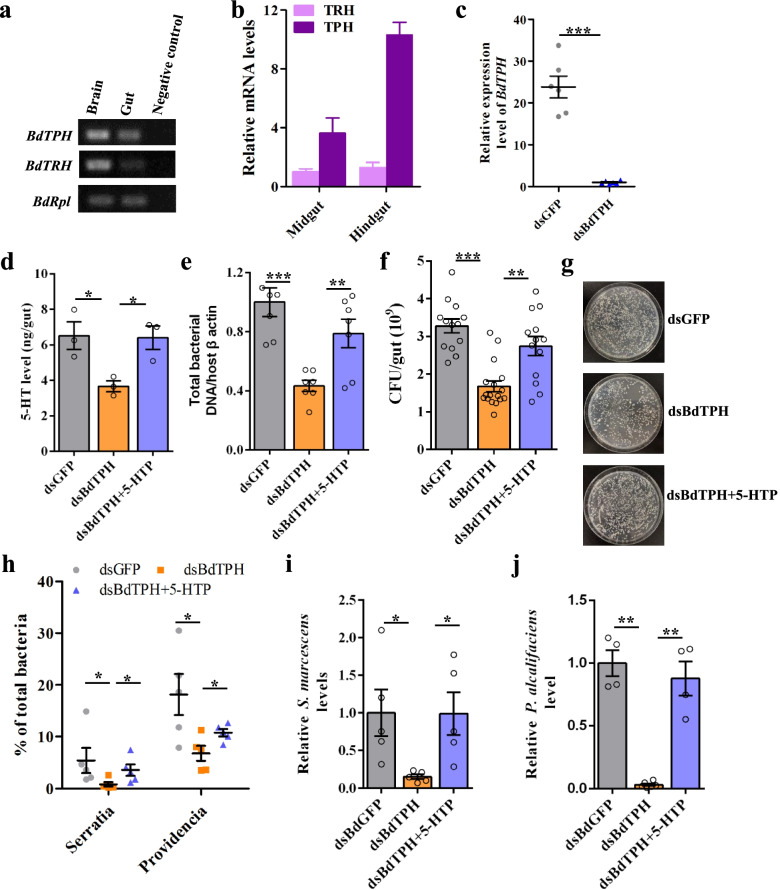
*BdTPH* contributes to the maintenance of gut microbiota in *B. dorsalis*. **a** Expression of *TPH* and *TRH* in the *B. dorsalis* gut. *RpL32* was used as an internal reference gene. **b** Relative expression levels of *BdTPH* and *BdTRH* in the midgut and hindgut of *B. dorsalis* (*n* = 3). **c** Gut *BdTPH* silencing efficiency in *B. dorsalis* at 96 h postinjection with 2.0 μg of ds*GFP* or ds*BdTPH*. Data were normalized to expression levels in ds*GFP*-treated flies. **d** Effect of *BdTPH* knockdown on gut 5-HT level by HPLC detection post dsRNA injection. **e**–**g** Effect of *BdTPH* silencing on gut total bacterial load by **e** 16S rRNA quantification using real-time PCR and **f**, **g** colony-forming unit assay. **h** Relative abundance of the genera *Serratia* and *Providencia* postinjection of ds*BdTPH* and ds*GFP* by 16S rDNA sequencing. **i** *BdTPH* knockdown decreased the load of *S. marcescens* and **j** *P. alcalifaciens* in the gut of *B. dorsalis*. To restore the 5-HT level, *BdTPH*-silenced flies were treated with 1-mM 5-HTP by feeding after dsRNA injection. Data were analyzed using two-tailed unpaired *t*-test. Error bars indicate ± s.e.m.; *p < 0.05, **p < 0.01, ***p < 0.001. All results represent at least two independent experiments

On the basis of the above results, 5-HT was hypothesized to mediate insect gut microbiome homeostasis. Tryptophan hydroxylase is the rate-limiting enzyme for 5-HT biosynthesis in insects, and the different isoenzymes TPH and TRH mediate non-neuronal and neuronal 5-HT biosynthesis, respectively [23, 24]. The transcripts of both *TPH* and *TRH* were detected in the *B. dorsalis* intestinal tract (Fig. 2a), although the mRNA level of *TPH* was much higher than that of *TRH* (Fig. 2b). In addition, more *TPH* transcripts were expressed in the hindgut than in the midgut (Fig. 2b).

To validate the role of TPH in controlling the gut microbiome, flies were injected with *BdTPH* double-stranded RNA (dsRNA) to silence the expression of the *BdTPH* gene. Flies treated with *green fluorescent protein* (*GFP*) dsRNA were the controls. Strong knockdown effect, approximately 97%, was observed post RNA-interference (RNAi) treatment (Fig. 2c). Compared with the *GFP* dsRNA-treated flies, the flies treated with *BdTPH* dsRNA had decreased levels of 5-HT in the gut, whereas treatment with 1-mM 5-HTP recovered the level of 5-HT (Fig. 2d). The diminished expression of *BdTPH* markedly inhibited the proliferation of intestinal tract bacteria (Fig. 2e–g). However, treatment with 5-HTP fully rescued the ds*-BdTPH*-induced phenotype (Fig. 2e–g). *BdTPH* dsRNA treatment changed the composition of the gut microbial community (Additional file 1: Fig. S4). Compared with the *GFP* dsRNA-treated controls, the relative abundance of the genus *Serratia* and *Providencia* decreased significantly after ds*-BdTPH* treatment (Fig. 2h and S4). However, by treating *BdTPH*-silenced flies with 1-mM 5-HTP, the reduction reversed dramatically (Fig. 2h, Additional file 1: Fig. S4). The abundances of *S. marcescens* and *P. alcalifaciens* in the *B. dorsalis* intestinal tract were then determined, and the loads of both species of bacteria decreased significantly, compared with those of the controls (Fig. 2i, j).

Next, the role of TRH in the maintenance of commensal gut microorganisms in *B. dorsalis* was examined. Intestinal tract *BdTRH* transcripts decreased significantly post RNAi treatment (Additional file 1: Fig. S5a). The ds-*BdTRH* silencing reduced midgut *BdTRH* mRNA levels but did not affect the amount of gut bacteria (Fig. S5a and b). There were no significant differences in the load of *S. marcescens* in the *B. dorsalis* intestinal tract between ds-*BdTRH*-treated flies and ds-*GFP*-treated controls (Additional file 1: Fig. S5c).

Collectively, the results indicated that the 5-HT controlled *B. dorsalis* gut microbiome homeostasis mainly via the peripheral serotonin biosynthetic enzyme TPH.

### Serotonin regulates gut microbiome homeostasis by controlling Duox expression in B. dorsalis

In *Drosophila*, two mechanisms that maintain gut microbiome homeostasis are Duox-dependent ROS [14, 15] and IMD pathway-produced antimicrobial peptides (AMPs) [16]. To investigate the molecular mechanism by which 5-HT regulates gut microbiome proliferation, the effect of 5-HT on ROS production and AMP expression was determined.

**Fig. 3:**
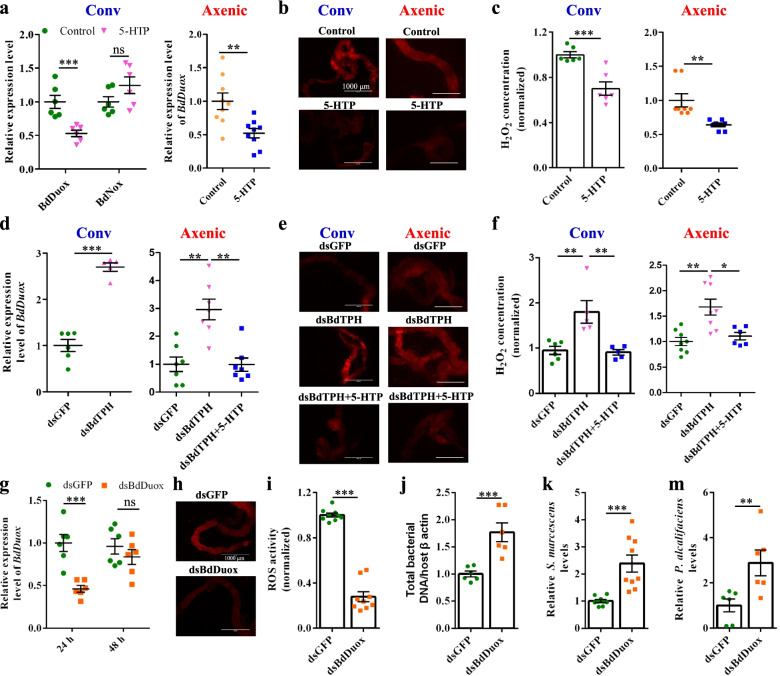
Serotonin controls gut *BdDuox* expression in *B. dorsalis*. **a–c** Effects of 5-HTP treatment on gut *BdDuox* and *BdNox* expression (**a**) and ROS activity (**b**, **c**) in conventionally colonized and axenic flies. ROS activity was measured by DHE (**b**) and H_2_O_2_ assay (**c**). Scale bars in **b** and **e** represent 1000 μm. Data of H_2_O_2_ level were normalized to controls. **d**–**f** Regulation of both *BdDuox* expression (**d**) and ROS activity (**e**, **f**) in the guts of conventionally colonized and axenic flies post ds*BdTPH* treatment. ROS activity was detected at 96 h post dsRNA injection. Data of H_2_O_2_ level were normalized to ds*GFP*-treated controls. To restore the 5-HT level, *BdTPH*-silenced flies were treated with 1-mM 5-HTP by feeding after dsRNA injection. **g**–**i** Gut *BdDuox* silencing efficiency determination in *B. dorsalis* by real-time PCR (**g**) and ROS activity (**h**, **i**). ROS activity was detected by DHE (**h**) and H_2_O_2_ assay (**i**) at 24 h post dsRNA injection. Scale bars in **h** represent 1000 μm. Data of H_2_O_2_ level were normalized to ds*GFP*-treated controls. **j**–**m** Effect of *BdDuox* silencing on gut total bacterial load (**j**) and the burden of *S. marcescens* (**k**) and *P. alcalifaciens* (**m**) at 24 h post dsRNA injection. Two-tailed unpaired *t*-test was performed in **a**, **c**, **d**, **g**, **i**, **j**, **k**, and **m**; one-way ANOVA followed by Tukey’s multiple comparison test for **f**. Conv, conventionally colonized flies; Axenic, axenic flies. Error bars indicate ± s.e.m.; *p < 0.05, **p < 0.01, ***p < 0.001. Results represent at least two independent experiments

In *Drosophila* intestine, ROS are produced and regulated by *Duox* [15] or nicotinamide adenine dinucleotide phosphate (NADPH) oxidase (*Nox*) genes [25]. Only one *Duox* gene and one *Nox* gene were identified in the genome of *B. dorsalis*, consistent with the findings in *Drosophila melanogaster* [15]. Compared with controls, the mRNA level of intestinal tract *BdDuox* in 5-HTP-treated flies was significantly downregulated by approximately twofold. However, 5-HTP had no effect on *BdNox* expression in conventionally colonized flies (Fig. 3a). To exclude the possibility that *Duox* has been activated by some microbiota, axenic flies were generated with antibiotics. The 5-HTP decreased *BdDuox* expression was observed in axenic fly guts (Fig. 3a). The reduced expression of *BdDuox* in 5-HTP-treated flies was consistent with the weaker ROS signal (Fig. 3b) and lower hydrogen peroxide (H_2_O_2_) production in the intestinal tract (Fig. 3c) of both conventionally colonized and axenic flies. Next, the regulation of *BdDuox* in the gut of *BdTPH*-silenced flies was validated by qPCR. Strong knockdown effect of *BdTPH* was observed post RNAi treatment in axenic adult *B. dorsalis* (Additional file 1: Fig. S6). The mRNA level of *BdDuox* was significantly upregulated post *BdTPH* silencing, compared with the *GFP* dsRNA-treated controls (Fig. 3d). In axenic flies, 5-HTP recovered *BdDuox* expression post *BdTPH* silencing (Fig. 3d). Consistent with this finding, ROS activity, as measured by dihydroethidium (DHE) (Fig. 3e) and H_2_O_2_ assay (Fig. 3f), increased consistently in the gut of *BdTPH*-silenced flies. The 5-HTP restored the ROS activity of both conventionally colonized and axenic flies post ds-*BdTPH* treatment (Fig. 3e, f). The knockdown of the *BdTRH* gene had no significant effect on *BdDuox* expression (Additional file 1: Fig. S7).

To validate the role of ROS in the control of gut bacteria, *BdDuox* expression was silenced by dsRNA injection. The ds-*BdDuox* silencing reduced intestinal tract *BdDuox* mRNA levels by 54% at 24 h post injection (Fig. 3g). Compared with the *GFP* dsRNA-treated controls, a weaker ROS signal (Fig. 3h) and lower H_2_O_2_ concentration (Fig. 3i) were detected in the intestinal tract of *BdDuox*-silenced flies. The *BdDuox* knockdown markedly promoted the proliferation of intestinal tract bacteria (Fig. 3j), including *S. marcescens* and *P. alcalifaciens* (Fig. 3k, m).

To assess changes in the expression profiles of *IMD*, *Relish*, and four effector genes encoding AMPs, including one *Diptercin* gene and three *Cecropin* genes, qPCR was used. None of the tested genes was differentially expressed between ds-*BdTPH*-treated flies and ds-*GFP-*treated controls (Additional file 1: Fig. S8).

Thus, the data indicate that serotonin regulates gut microbiome homeostasis by controlling *Duox* expression and therefore ROS activity.

### Serotonin-mediated Duox expression in response to gut bacteria in B. dorsalis

**Fig. 4:**
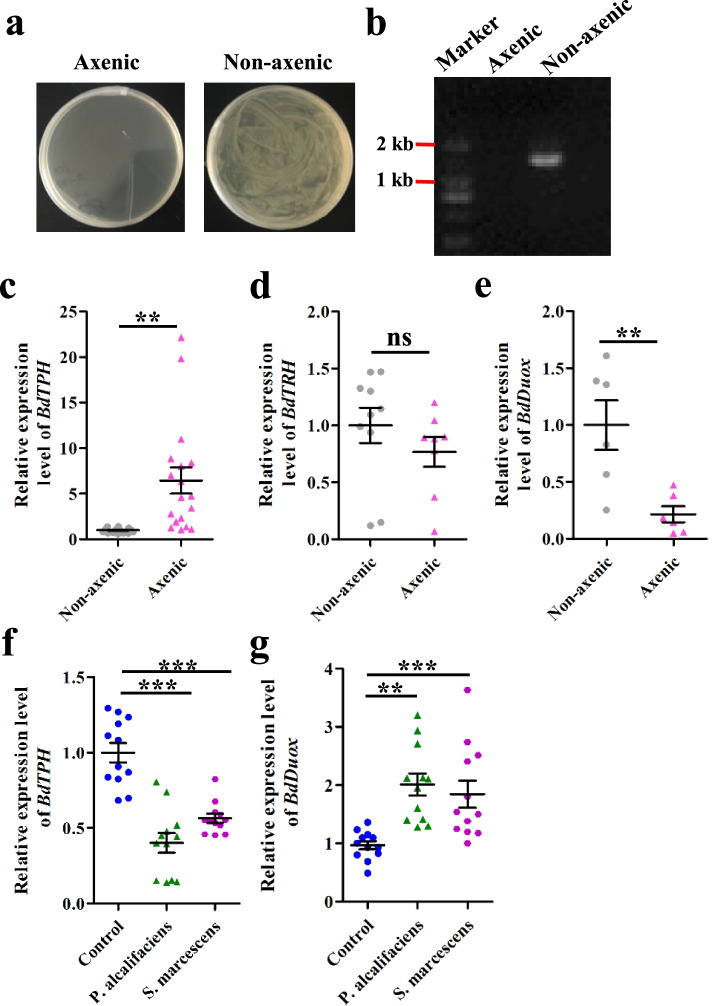
Serotonin biosynthesis in response to gut bacteria in *B. dorsalis*. **a**, **b** The efficacy of elimination of gut bacteria confirmed by culturing *B. dorsalis* gut homogenates on LB agar plates (**a**) and by performing PCR analysis (**b**) on gut homogenates using universal 16S rRNA gene primers. **c**–**e** Relative expression levels of *BdTPH*, *BdTRH*, and *BdDuox* in the gut of nonaxenic and axenic flies. **f**, **g** Gene abundance of *BdTPH* and *BdDuox* after the bacteria were introduced into the guts of antibiotic-treated flies. Two-tailed unpaired *t*-test was performed for **c–g**. Error bars indicate ± s.e.m., **p < 0.01, *p < 0.05, and ns means no significant difference. All results were repeated in at least two independent experiments, with three biological replicates

Our results indicate that the peripheral serotonin is a key factor maintaining *B. dorsalis* gut microbiome homeostasis. Next, we assessed whether gut bacteria affect 5-HT biosynthesis. Axenic flies were generated by antibiotic treatment (Fig. 4a, b). Expression of *BdTPH* was significantly higher in the intestinal tract of axenic flies than in nonaxenic flies (Fig. 4c). No significant difference of *BdTRH* transcripts were detected between axenic and nonaxenic flies (Fig. 4d). The mRNA level of *BdDuox* was lower in axenic flies than in nonaxenic flies (Fig. 4e). We then examined whether the expression of *BdTPH* is correlated with specific members of the fly gut microbiome. We isolated *P. alcalifaciens*, *S. marcescens*, and *Klebsiella sp.* from *B. dorsalis* guts and these bacteria have been identified as cultivable gut commensals. Among these bacteria, both *P. alcalifaciens* and *S. marcescens* robustly proliferated in response to 5-HT elevation. We next cultured these bacteria and orally fed them to antibiotic-treated flies. Gut *BdTPH* gene was downregulated by oral introduction of *P. alcalifaciens* and *S. marcescens* (Fig. 4f), while *BdDuox* was upregulated after ingestion of *P. alcalifaciens* and *S. marcescens* (Fig. 4g). The gut *BdTPH* mRNA level of *Klebsiella sp.* treated flies has no significant difference from that of controls (Additional file 1: Fig. S9). These data suggest that the 5-HT regulated *Duox* expression may be stimulated by specific bacterium.

### Serotonin controls Duox expression and gut microbiome load in A. aegypti

Mosquito-borne diseases constitute a large portion of infectious diseases, causing more than 700,000 deaths annually. Aedes mosquito-transmitted diseases, such as dengue, Zika, yellow fever, and chikungunya, have re-emerged recently and remain a public health threat worldwide [26]. The gut commensal microbiome has complicated roles in arboviral infection and transmission in vector mosquitoes [20, 27]. An understanding of the molecular mechanisms involved in the control of midgut microbiota could help in the development of new tools to reduce pathogen transmission. In *Aedes aegypti*, reduction midgut ROS levels results in dysbiotic proliferation of the intestinal microbiota [28]. The present study revealed 5-HT regulated gut ROS levels and promoted the proliferation of *Serratia*, which is involved in mosquito viral infection [19, 29]. Therefore, whether 5-HT regulates the midgut microbiota of the vector mosquito *A. aegypti* was investigated.

**Fig. 5:**
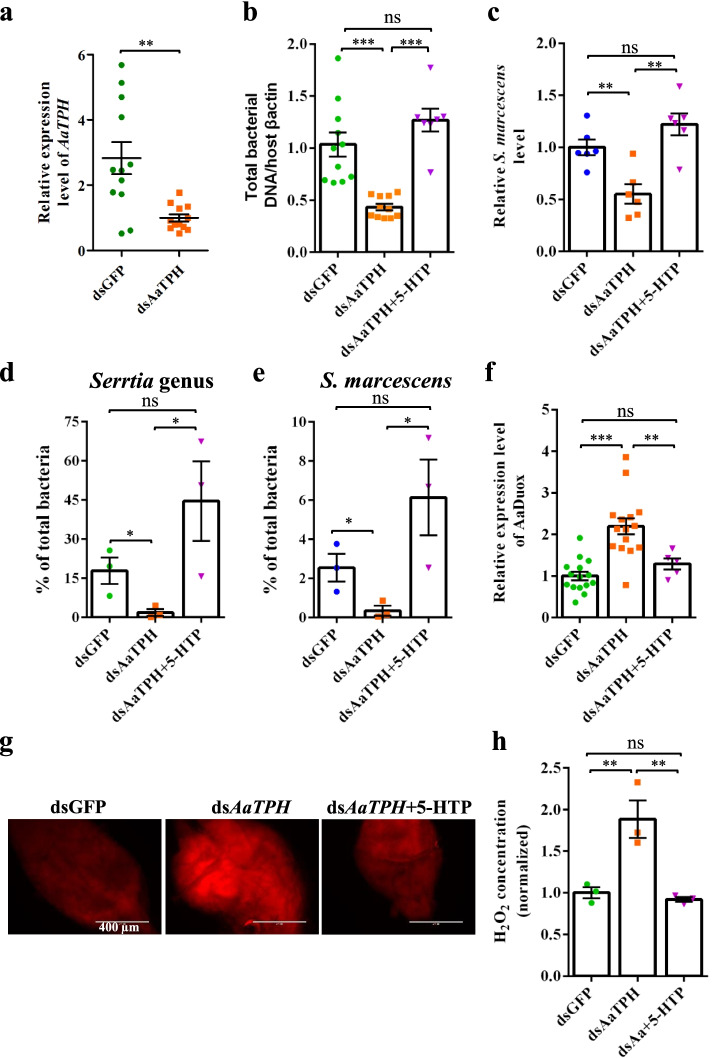
Serotonin maintains gut microbiome homeostasis by controlling *AaDuox* expression in *A. aegypti*. **a** Midgut *AaTPH* silencing efficiency at 96 h post injected with 1.2 ug of dsGFP or ds*AaTPH*. **b**, **c** Effect of *AaTPH* silencing on **b** midgut total bacterial load and **c** *S. marcescens*. **d**, **e** *AaTPH* knockdown decreased the relative abundance of **d** *Serrtia* genus and **e** *S. marcescens* in the midgut of *A. aegypti*. **f**–**h** Regulation of both **f** *AaDuox* expression and **g**, **h** ROS activity in the midguts of *AaTPH*-silencing mosquitoes. The *GFP* dsRNA-treated mosquitoes served as controls. ROS activity was detected by **g** DHE and **h** H_2_O_2_ assay post dsRNA injection. Scale bars in **g** represent 400 μm. Data of H_2_O_2_ level are normalized to *dsGFP*-treated controls. Two-tailed unpaired *t*-test was performed for **a**; one-way ANOVA followed by Tukey’s multiple comparison test for **b**, **c**, **d**, **e**, **f,** and **h**. Error bars indicate ± s.e.m., ***p < 0.001, **p < 0.01, *p < 0.05, and ns means no significant difference

The *TPH* gene was identified in the genome of *A. aegypti*. The *AaTPH* gene was knocked down via thoracic microinjection of dsRNA into *A. aegypti*. Compared with controls, the midgut mRNA level of *AaTPH* was significantly downregulated post dsRNA injection (Fig. 5a). Midgut bacterial load was significantly reduced in the *AaTPH*-RNAi mosquitoes compared with that in the midgut of *GFP*-RNAi controls (Fig. 5b, c). Treatment with 5-HTP rescued the *AaTPH*-RNAi-induced effects on gut bacterial burden (Fig. 5b, c). Then, the effect of 5-HT on the composition of midgut microbes in *A. aegypti* was assayed. Consistent with the findings in *B. dorsalis*, with the knockdown of *AaTPH*, the relative abundance of the genus *Serratia* decreased significantly, including that of the species *S. marcescens* (Fig. 5d, e). The 5-HTP rescued the proportion of *Serratia* in the gut bacterial community of *AaTPH*-RNAi mosquitoes (Fig. 5d, e).

Next, whether 5-HT controlled *Duox* expression and ROS activity in *A. aegypti* was determined. Consistent with the observation in *B. dorsalis*, *AaDuox* transcripts increased significantly by approximately twofold in *AaTPH-*RNAi mosquitoes compared with those in *GFP*-RNAi controls (Fig. 5f). However, by treating *AaTPH*-silenced mosquitoes with 1 mM 5-HTP, the increase reversed dramatically (Fig. 5f). Consistent with this finding, ROS activity, as measured by DHE (Fig. 5g) and H_2_O_2_ assay (Fig. 5h), increased consistently in the gut of *AaTPH*-silenced flies. These data suggested that *AaTPH* regulated midgut microbiota by regulating *Duox* expression in *A. aegypti*.

## Discussion

Commensal microbes colonize the gut epithelia of virtually all animals, and changes in commensal populations can lead to dysbiosis, which is associated with numerous pathologies [30]. The mucosal immune system uses multiple complex mechanisms to maintain a balance between preserving beneficial microbiota and eliminating pathogens. In a previous study, Qi et al. showed that 5-HT regulates insect hemocyte immune responses [31]. In the current study, the authors show that 5-HT modulates gut commensal populations via the mucosal immune system.

In this study, 5-HT controlled the gut bacterial load in *B. dorsalis* and *A. aegypti*. Whereas an elevated level promoted the amount of gut bacteria, when peripheral 5-HT biosynthesis was reduced via knockdown of the *TPH* gene, the gut bacterial burden decreased. In mammals, the gastrointestinal tract is an important site for 5-HT biosynthesis. Elevated levels of intestinal lumenal 5-HT increase the relative abundance of spore-forming members of the gut microbiota and improve the ability of *Turicibacter* to competitively colonize the intestine [7]. In *B. dorsalis*, 5-HT increased the relative abundance of *Serratia* and *Providencia* in the midgut and hindgut, respectively. This result suggests that the serotonin–Duox axis-based regulation pathway is specific for particular bacteria.

*Serratia* and *Providencia* are ubiquitous gram-negative bacteria in the family *Enterobacteriaceae* that are often opportunistic pathogens. *Serratia marcescens* is a widely recognized insect pathogen [32–34], and in *D. melanogaster*, *P. alcalifaciens* is highly virulent, causing from 90 to 100% mortality in infected flies [22]. In this study, with 5-HT, the relative abundance of gut *S. marcescens* and *P. alcalifaciens* in *B. dorsalis* increased. The 5-HT-induced gut microbiota dysbiosis may explain why the continuous ingestion of 5-HTP led to insect death. In mosquitoes, the gut commensal microbiome plays complicated roles in arboviral infection and transmission. Gut commensal bacteria limit viral infections by priming host immune surveillance and by secreting microbial metabolites [27, 35]. Nevertheless, the midgut bacterium *Serratia odorifera* enhances viral infection in *Aedes* [19] and *Anopheles* mosquitoes [29]. The gut commensal bacterium *S. marcescens* increases the susceptibility of field *A. aegypti* to dengue virus [20] and promotes fungal killing of mosquitoes [36]. In this study, 5-HT increased the relative abundance of *Serratia* in the midgut of *A. aegypti*. On the basis of these results, new ways to reduce virus transmission may be possible via modulation of the 5-HT signaling pathway in mosquitoes.

Insect feeding behavior may affect gut microbiome. Previous studies reveal that 5-HT inhibits insect feeding. In *Camponotus mus* ants, orally administered 5-HT diminished sucrose intake [37]. Serotonin injection decreased feeding in the flesh fly *Neobellieria bullata* [38]. In adult foraging honeybees, 5-HT inhibits feeding in the brain, but general elevation of 5-HT in the bee’s haemolymph does not affect food intake [39]. In this study, orally administered 5-HTP diminished the food intake in *B. dorsalis* (Additional file 1: Fig. S10). Therefore, 5-HT-induced gut bacteria load may be not from food intake. In the present study, 5-HT regulated gut microbiome homeostasis via regulating *Duox* expression. Several studies show that Duox-mediated production of ROS is a major immune mechanism regulating insect gut–microorganism homeostasis [14, 15, 40]. Duox-mediated ROS are required for routine control of *Erwinia carotovora carotovora 15*, a gut pathogen in *Drosophila* [15]. A reduction in ROS levels in the midgut allows proliferation of intestinal microbiota in *A. aegypti* [28]. Duox-mediated ROS also play an important role in regulating homeostasis and the composition of the gut bacterial community in *B. dorsalis* [41]. Consistent with previous findings, the results of present study revealed that decreasing ROS levels via knockdown of the *Duox* gene caused gut microbiota dysbiosis in *B. dorsalis*. With typical microbial burdens, such as those found in commensal–gut interactions, both the expression and activity of *Duox* are tightly restricted to a level that allows healthy gut–microorganism interactions, thereby precluding any pathophysiological effects on the gut epithelia. The Gαq–phospholipase C-β (PLC-β) signaling modulates Duox activity through Ca^2+^ mobilization [14]. The induction of *Duox* gene expression is limited in an off-state status by PLC-β-mediated mitogen-activated protein kinase (MAPK) P38 dephosphorylation in the *Drosophila* gut [40]. Therefore, the basal expression and activation of *Duox* are essential to manage symbiotic microorganisms under healthy conditions in the insect gut. In this study, 5-HT decreased *Duox* expression and ROS activity. In vertebrates, 5-HT modulates MAPK activity [42, 43] and functions to elevate intracellular ROS levels in peripheral cells [44, 45]. These findings suggest that although 5-HT regulation of ROS level is most likely conserved, a modified molecular mechanism leads to suppression of ROS in the insect gut instead of promotion. Insect 5-HT receptors are G protein-coupled receptors that regulate intracellular cyclic adenosine monophosphate (cAMP) or Ca^2+^ level via activation of protein kinase A (PKA) or C (PKC), respectively [46]. In mosquitoes, heme-mediated downregulation of gut ROS occurs through PKC activation [28]. Therefore, 5-HT might modulate *Duox* expression via its receptors. Insect 5-HT receptors are classified as 5-HT_1A/1B_, 5-HT_2A/2B_, and 5-HT_7_ on the basis of sequence similarities with their counterparts in vertebrates. In future work, the roles of MAPK, PKA, and PKC in serotonin-mediated downregulation of ROS should be investigated, and the receptor that modulates *Duox* expression and ROS activity determined.

## Conclusions

In conclusion, an important role of 5-HT in insect gut microbiome homeostasis was discovered. The peripheral 5-HT biosynthetic enzyme TPH mediated gut bacterial load via *Duox* expression and ROS activity. ROS, an important immune effector in insect gut, may impose substantial immunopathological costs. Excessive ROS levels leads to a shorter lifespan and increased apoptosis in the gut of *D. melanogaster* [40]. 5-HT regulated inhibition of basal ROS production in Dipteran may reduce tissue damage. The increased gut symbiotic bacterial load may improve the tolerance to pathogens. These findings provide new insights into gut–microbe interactions and may lead to new strategies to control pests and prevent transmission of vector-borne disease.

## Methods

### Insect rearing

*Bactrocera dorsalis* was reared at 27 °C and 75% relative humidity under a 14:10 h light:dark photoperiod. Adults were fed an artificial diet consisting of yeast extract and dry sugar mixed at a 1:1 ratio (w/w) and housed in transparent plastic cages. *Aedes aegypti* was reared at 26 °C and 80% relative humidity under a 16:8 h light: dark photoperiod. Mosquito larvae were raised on fish food. Male and female adult mosquitoes were maintained in a cage with unlimited access to cotton balls moistened with a 10% sterile sucrose solution. Five-day-old female mosquitoes were used in the experiments.

### Drug treatment and serotonin measurement

Serotonin was quantified using reverse-phase high-performance liquid chromatography (HPLC). To develop dose–response curves, 5-day-old *B. dorsalis* adults, a mix of the same number of males and females, were treated with sterile 5% sugar solution with 5-HTP mixed in at concentrations ranging from 0 to 10 mM. Three replicates of 20 flies were tested at each concentration. After 4 days of treatment, intestinal tracts (including midgut and hindgut) were dissected in a petri dish kept cold on ice. Twenty intestinal tracts were stored in 200 μl of phosphate buffered saline (PBS; 0.01 M, pH 7.4) and homogenized using beads. Then, an equal volume of 4% perchloric acid was added. The tubes were vortexed to extract 5-HT. The homogenates were centrifuged at 14,000×*g* for 15 min at 4 °C. The supernatants were transferred to 1.5-ml Eppendorf tubes and stored at −80 °C until HPLC analysis. Twenty microliters of supernatant was automatically loaded into an Inlet Systems (1260 HPLC-UV system, Agilent Technologies, CA, USA) with a Zorbax SC-C18 column (4.6 × 250 mm, 5 μm; Agilent Technologies). The HPLC analysis for the samples were conducted as the following conditions: the mobile phase consisted of 0.1% formic acid aqueous solution and methanol (93:7 v/v), at a flow rate of 1.0 mL/min. UV detection was set at 254 nm, and column temperature was 30 °C. Total run time was 15 min, and retention time for serotonin was 5.2 min. The serotonin standard was purchased from Sigma-Aldrich (St. Louis, MO, USA). The serotonin concentration in the intestinal tracts was quantified by the HPLC-external standard method (standard curve: *y* = 6.0716*x* + 3.3193, *R*^2^ = 0.9977, where *x* is the peak height of the serotonin standard and *y* is the serotonin concentration). The identity of detected serotonin in samples was confirmed by comparing their retention time and UV spectra with those of standards. Experiments were performed with three biological replicates.

To rescue the 5-HT level in *BdTPH*-RNAi flies, *TPH* knockdown flies continuously ingested 1-mM 5-HTP added in sterile 5% sucrose solution for 4 days after dsRNA injection. The methods for 5-HT detection were the same as described above. Experiments were performed with three biological replicates, each replicate containing 20 flies.

### Quantification of culturable bacteria in the intestinal tract of B. dorsalis

The culturable bacteria of the intestinal tract of *B. dorsalis* were quantified by a colony-forming unit assay. To isolate bacteria from the gut, *B. dorsalis* adults were surface-sterilized in 75% ethanol for 3 min and then rinsed three times in sterile PBS (pH 7.4). The intestinal tracts were dissected under aseptic conditions and were ground in 200 μl of sterile PBS. The homogenates of the guts were serially diluted 1000 times with PBS and plated onto Luria–Bertani medium agar plates. After overnight culture at 37 °C, the bacteria on the plate were counted to calculate the number of bacteria per insect.

### Quantification of gut bacteria by quantitative PCR

The genomic DNA from the intestinal tracts of *B. dorsalis* or the midguts of *A. aegypti* was extracted using an EZNA® Soil DNA Kit (Omega Bio-Tek, Inc., Norcross, GA, USA) according to the manufacturer’s instructions. Bacterial quantitation by qPCR was performed on genomic DNA using universal eubacteria primers to amplify 16S ribosomal RNA (rRNA) fragments [41]. We use *S. marcescens* [47] and *P. alcalifaciens* [48] specific primers to quantity those bacteria by qRT-PCR respectively. The primers are shown in Additional file 2: Table S1. The qPCR was conducted on a Stratagene Mx3000P thermal cycler (Agilent Technologies, Wilmington, DE, USA) with TB Green Premix Ex Taq II (Tli RNase H Plus; Takara Bio, Otsu, Japan). Thermal cycling conditions were the following: 95 °C for 30 s, 40 cycles at 95 °C for 5 s, and 60 °C for 34 s. Three technical replicates were analyzed for each sample. No-template negative controls were included in each run to detect possible contamination or carryover. The housekeeping *β-actin* gene was used as an endogenous control.

### DNA sample preparation and deep sequencing

The extraction of total bacterial DNA from the guts of *B. dorsalis* or *A. aegypti* was the same as described above. Three replicates were prepared for 1-mM 5-HTP-treated *B. dorsalis* and the controls. Each replicate contained 10 flies, and the midgut and hindgut were dissected separately. Five replicates were prepared for dsRNA-treated *B. dorsalis*, and each replicate contained 10 flies. Three replicates were prepared for dsRNA-treated *A. aegypti*. Each replicate contained 20 mosquitoes, and the midguts were dissected. Approximately 465 bp of the V3+V4 region of the bacterial 16S rDNA gene was amplified by PCR. The following primers were used: F, CCTACGGGNGGCWGCAG; R, GGACTACHVGGGTATCTAAT. PCRs were performed in triplicate in 50 μL mixtures containing 5 μL of 10 × KOD Buffer, 5 μL of 2.5 mM dNTPs, 1.5 μL of each primer (5 μM), 1 μL of KOD Polymerase, and 100 ng of template DNA. Reactions consisted of one cycle at 95 °C for 2 min, followed by 27 cycles at 98 °C for 10 s, 62 °C for 30 s, and 68 °C for 30 s and a final extension at 68 °C for 10 min. The PCR products were checked using 2% agarose gel electrophoresis, purified using an AxyPrep DNA gel extraction kit (Axygen Biosciences, Union City, CA, USA) according to the manufacturer’s instructions, and quantified using fluorescence quantitation (QuantiFluor -TM, Promega, USA).

Purified amplicons were pooled in equimolar concentrations and paired-end sequenced (2 × 250) on an Illumina platform according to standard protocols. Data analysis was performed according to Huang et al. [49]. Raw reads were removed if they contained more than 10% of unknown nucleotides (N) or fewer than 80% of bases with quality (Q-value) > 20. Paired-end clean reads were merged as raw tags using FLASH (v1.2.11) with a minimum overlap of 10 bp and mismatch error rate of 2%. Noisy sequences of raw tags were filtered with the QIIME (V1.9.1) pipeline under specific filtering conditions to obtain high-quality clean tags. Clean tags were searched against the reference database (http:// drive5.com/uchime/uchime_download.html) to perform reference-based chimera checking using the UCHIME algorithm (http://www.drive5.com/usearch/manual/uchime_algo.html). All chimeric tags were removed, and the remaining tags were subjected to further analysis. Tags were clustered into operational taxonomic units (OTUs) of ≥97% similarity using the UPARSE pipeline [50]. The tag sequence with the highest abundance was selected as a representative sequence within each cluster. The representative sequences were classified into organisms with a naive Bayesian model using the RDP classifier (version 2.2) based on the SILVA database (https://www.arb-silva.de/).

### Survival assay

To obtain axenic flies, 5-day-old adults, a mixed of the same number of males and females, were feeding with 5% sucrose solution containing 50 μg/ml tetracycline, 100 μg/mL penicillin-streptomymin, 150 μg/mL gentamycin, and 150 μg/mL rifampicin for 4 days. Controls were fed 5% sterile sugar solution. Flies were then treated with sterile water without antibiotic for 1 day to allow the antibiotics to be metabolized. The flies were decontaminated in 70% ethanol and rinsed in sterile PBS, and the intestinal tracts were dissected under aseptic conditions. Removal of the microorganisms was confirmed by performing PCR analysis using universal 16S rRNA gene primers. To detect the effect of 5-HTP on *B. dorsalis* mortality, flies were treated with 1-mM 5-HTP in 5% sterile sugar solution daily. Control flies were fed 5% sterile sugar solution only. Experiments were performed independently three times, with three biological replicates, each replicate containing 20 flies. The survival rate was recorded every 24 h.

To determine the effect of gut-isolated bacteria on the *B. dorsalis* survival rate, *S. marcescens* and *P. alcalifaciens* were cultured separately overnight at 37 °C. After centrifugation at 7500 rpm for 10 min, bacterial pellets were washed twice with PBS. Then, the pellets were resuspended in sterile 5% sugar solution, reaching OD _600_ = 1.5. To infect the flies, 5-day-old *B. dorsalis* adults, a mix of the same number of males and females, were continuously fed the resulting solution. Control flies were fed a diet supplemented with sterile 5% sucrose only. Surviving flies were counted daily. Experiments were performed independently twice, with three biological replicates, each replicate containing 20 flies.

### Gene cloning

Total RNA was isolated from the guts of 20 adults using TRIzol reagent (Invitrogen, Carlsbad, CA, USA) and then treated with RQ1 DNase I (Promega, Madison, WI, USA) to eliminate genomic DNA. Single-strand cDNA synthesized from total RNA aliquots (1 μg) using a RevertAid First Strand cDNA Synthesis Kit (Thermo Scientific, Waltham, MA, USA) was used as a template for the PCR. The primers are listed in Additional file 2: Table S1. The PCR was performed using Q5 High-Fidelity DNA Polymerase (New England Biolabs), according to the manufacturer’s instructions. To determine their sizes, amplification products were separated by electrophoresis on 1.0% agarose gels. Purified PCR products were then cloned into a pEASY-Blunt Zero Cloning Vector (TransGen, Beijing, China), following the manufacturer’s instructions, before being sequenced.

### Reverse-transcription quantitative PCR analysis

To analyze gene expression level in the intestinal tract of *B. dorsalis*, the guts of 5-day-old adults, a mix of the same number of males and females, were dissected. Experiments were performed independently at least twice, with three biological replicates, each replicate containing 10 flies. To analyze gene expression in *A. aegypti*, midguts of 5-day-old females were dissected. Experiments were performed independently at least twice, with three biological replicates, each replicate containing 20 mosquitoes. RNA was extracted using TRIzol reagent (as described above). The purity of the extracted RNA was assessed spectrophotometrically by measuring the OD_260/280_ ratio and an OD_260/280_ of 1.8–2.0 indicates good quality RNA. RNA integrity was measured via electrophoresis on a formaldehyde agarose gel. The RNA aliquots, 1 μg, were reverse transcribed to cDNA using a PrimeScript™ RT reagent Kit with gDNA Eraser (Takara) according to the manufacturer’s instructions. Biosynthesized cDNA was used as a template in the RT-qPCR, conducted on a Stratagene Mx3000P thermal cycler (Agilent Technologies, Wilmington, DE, USA) with TB Green Premix Ex Taq II (Tli RNase H Plus) (Takara Bio, Otsu, Japan). Thermal cycling conditions were the following: 95 °C for 30 s, 40 cycles at 95 °C for 5 s, and 60 °C for 34 s. Three technical replicates were analyzed for each sample. No-template negative controls were included in each run to detect possible contamination or carryover. A series of gene-specific primers were designed for the RT-qPCR using the software Primer 3 (http://bioinfo.ut.ee/primer3-0.4.0/) (Additional file 2: Table S1). The specificity of RT-qPCR reaction products was established via electrophoresis on 1.0% agarose gels before sequencing. The transcript levels of different genes were quantified using the 2^−ΔΔCT^ method [51]. *α-tubulin* [52] and *RpL32* [41] were used as reference genes for gene expression analysis in *B. dorsalis*, due to their expression stability. The housekeeping *AaS7* gene was used as an endogenous control for *A. aegypti*. The gene expression of each sample was normalized to that of controls (taken as 1).

### dsRNA-mediated gene silencing

PCR-generated DNA templates were used to biosynthesize dsRNA, which contained T7 promoter sequences at each end. The primers used are listed in Additional file 2: Table S1. The amplicons were purified and verified with DNA sequencing. A MEGAscript T7 transcription kit (Ambion, Austin, TX, USA) was used to produce the specific dsRNA of each gene following the manufacturer’s instructions. As a negative control, green fluorescent protein (GFP) dsRNA was biosynthesized. The quality and size of the dsRNA products were verified by 1% agarose gel electrophoresis, and the dsRNA products were diluted with nuclease-free water to the final concentration of 4 μg/μl. The dsRNA, 2 μg, of the target gene or GFP control was injected into the thoracic hemocoel of 5-day-old *B. dorsalis* adults using a FemtoJet microinjection system (Eppendorf). Gene silencing experiments in *A. aegypti* were performed by injecting 1.2 μg of dsRNA into the thorax of cold-anesthetized 5-day-old females. The efficiency of dsRNA-mediated gene silencing was determined by RT-qPCR at 1 to 4 days after injection. Experiments were performed independently three times, with three biological replicates. Insects fed sterile sucrose solution.

### Antibiotic treatment of B. dorsalis and oral ingestion of bacteria

Ten-day-old *B. dorsalis* adults mixed with the same number of males and females were treated with 5% sucrose solution containing 50 μg/ml tetracycline, 100 μg/mL penicillin-streptomymin, 150 μg/mL gentamycin, and 150 μg/mL rifampicin for 4 days. The flies were then treated with sterile water without antibiotic for 1 day to allow the antibiotics to be metabolized. The flies were decontaminated in 70% ethanol and rinsed in sterile PBS, and the intestinal tracts were dissected under aseptic conditions. Removal of the microorganisms was confirmed by performing PCR analysis using universal 16S rRNA gene primers (Additional file 2: Table S1) and CFU assay. Dissected guts were used for qRT-PCR. Experiments were performed independently twice, with five biological replicates.

*Serratia marcescens*, *P. alcalifaciens*, and *Klebsiella sp.* were isolated from the *B. dorsalis* gut. They were cultured in Luria–Bertani broth at 37 °C for 16 h. The bacterial culture was then pelleted by centrifugation (3000×*g*, 5 min), washed twice in sterile PBS, and resuspended in 5% sterile sucrose solution ((optical density = 50). The bacterial suspension was provided to the axenic flies for 12 h. All supplies used in the fly feeding experiment were aseptic. The bacteria-fed flies were killed and the guts isolated. The dissected guts were used in RT-qPCR.

### In vivo detection of reactive oxygen species

The intestinal tracts (midgut and hindgut) of *B. dorsalis* and the midguts of mosquitoes were dissected in PBS containing the catalase inhibitor 3-amino-1,2,4-triazole (A8056, Sigma). Immediately after dissection, the guts were incubated with 2 μM dihydroethidium (DHE, D7008, Sigma) in PBS at room temperature for 30 min in the dark. The guts were then fixed with 4% paraformaldehyde for 30 min and incubated for an additional 30 min with 5% Triton X-100. Fluorescence images were collected using an EVOS FL Auto microscope (Life Technologies, Carlsbad, CA, USA) at 10× magnification.

### Measurement of hydrogen peroxide production

The production of H_2_O_2_ was determined according to [36]. A Hydrogen Peroxide Assay Kit (Beyotime Biotech, Shanghai, China) was used according to the manufacturer’s instructions. In this assay, H_2_O_2_ converts Fe^2+^ to Fe^3+^, which then complexes with xylenol orange dye to yield a purple product having an absorbance maximum at 560 nm, detectable by a spectrometer.

The insect guts were dissected in PBS with 2 mg/ml catalase inhibitor 3-amino-1,2,4-triazole (A8056, Sigma). Ten intestinal tracts were dissected for each biological replicate of *B. dorsalis*. Forty midguts were dissected for each replicate of *A. aegypti*. The dissected guts were homogenized in 200 μl of lysis buffer and centrifuged at 12,000×*g* at 4 °C for 5 min, and the supernatant was collected. Aliquots of 50 μl of supernatant and 100 μl of test solution from the hydrogen peroxide assay kit were incubated at room temperature for 20 min and measured immediately with a spectrometer at the wavelength of 560 nm. The concentration of H_2_O_2_ released was calculated according to a hydrogen peroxide standard curve. The measurement was repeated three times. Experiments were performed with three biological replicates.

### Statistical analyses

Statistical analyses were performed using Prism 5 (GraphPad Software). Two-tailed *t*-test was used for unpaired comparisons between two groups of data. For the comparison of three or more sets of data, one-way ANOVA was performed, followed by Tukey’s multiple comparison test. A value of *p* < 0.05 was considered to be statistically significant. Each experiment had three biological replicates.

## Supplementary Information

**Additional file 1: Fig. S1.** Schematic of 5-HT synthesis. Serotonin (5-HT) is synthesized in a two-step process. The amino acid tryptophan is first hydroxylated by Tryptophan hydroxylase (TPH). This is the rate-limiting step in 5-HT synthesis. In insects, two gene products, TRH and TPH, are capable of converting tryptophan into 5-hydroxytryptophan (5-HTP). In the second step, 5-HTP is decarboxylated to serotonin by Dopa decarboxylase (DDC). **Fig. S2.** Effect of 5-HTP treatment on midgut and hindgut bacterial composition in *B. dorsalis* at genus level. **Fig. S3.** Effect of 5-HTP treatment on the relative abundance of *S. marcescens* and *P. alcalifaciens* in the gut of *B. dorsalis*. (**a**) Phylogenetic analysis of the identified *S. marcescens* and *P. alcalifaciens* operational taxonomic units (OTUs) with 18 related bacteria. The evolutionary history was inferred by using the neighbor-joining method conducted in MEGA 6 software. A sequence of *Pseudomonas entomophila* was used as the outgroup. Bootstrap values (percentages of 1,000 tree replications) greater than 50% are displayed at the nodes. Sequence GenBank accession numbers are shown in parentheses. (**b**, **c**) 5-HTP treatment increased the relative abundance of (**b**) *S. marcescens* and (**c**) *P. alcalifaciens* in the gut of *B. dorsalis*. **Fig. S4.** BdTPH regulates gut bacterial composition in *B. dorsalis*. (**a**) Histogram showing changes at the genus level. (**b**) Relative abundance of bacteria at the genus level in *B. dorsalis* gut postinjection of *dsBdTPH* and *dsGFP* by 16S rDNA sequencing. To restore the 5-HT level, *BdTPH*-silenced flies were treated with 1-mM 5-HTP by feeding after dsRNA injection. **Fig. S5.** *BdTRH* knockdown does not affect the gut commensal microbiome of *B. dorsalis*. (**a**) Gut *BdTRH* silencing efficiency in *B. dorsalis* at 72 h postinjection with 1.5 μg of ds*GFP* or ds*BdTRH*. (**b**) Effect of *BdTRH* silencing on gut total bacterial load. (**c**) Effect of *BdTRH* knockdown on gut *S. marcescens* burden. The housekeeping β-actin gene was used as an endogenous control. **Fig. S6.** Gut *BdTPH* silencing efficiency in axenic *B. dorsalis*. (**a**-**b**) The efficacy of elimination of gut bacteria confirmed by culturing *B. dorsalis* gut homogenates on LB agar plates (**a**) and by performing PCR analysis (**b**) on gut homogenates using universal 16S rRNA gene primers. (**c**) The mRNA level of *BdTPH* in axenic *B. dorsalis* gut at 96 h postinjection with 2.0 μg of ds*GFP* or ds*BdTPH*. Data were normalized to expression levels in ds*GFP*-treated flies. **Fig. S7.** Effect of *BdTRH* silencing on gut *BdDuox* expression level in *B. dorsalis*. **Fig. S8.** Expression of six genes related to IMD pathway in the guts of *BdTPH*-silencing *B. dorsalis*. The *GFP* dsRNA-treated flies served as controls. Gene expression of each sample was normalized to that of controls (taken as 1). **Fig. S9.** Relative expression levels of *BdTPH* after the bacteria *Klebsiella sp.* were introduced into the guts of antibiotic-treated flies. **Fig. S10.** The effect of 1mM 5-HTP on liquid food consumption by *B. dorsalis*.

**Additional file 2: Table S1.** Primers used in this study.

## Data Availability

All the raw sequencing data for *B. dorsalis* and *A. aegypti* have been disposed in NCBI’s Sequence Read Archive (SRA) and are accessible through BioProject accession PRJNA735076 [53], PRJNA735433 [54], and PRJNA735435 [55]. Data supporting the findings of this work are available within the paper and its Supplementary Information files.
