# Supplementary material for: Serotonin modulates insect gut bacterial community homeostasis
Source: BMC Biol. 2022 May 13;20:105. doi: 10.1186/s12915-022-01319-x (PMC9103294; doi:10.1186/s12915-022-01319-x)
Supplement: Supplementary file 1 — Additional file 1: Fig. S1. Schematic of 5-HT synthesis. Serotonin (5-HT) is synthesized in a two-step process. The amino acid tryptophan is first hydroxylated by Tryptophan hydroxylase (TPH). This is the rate-limiting step in 5-HT synthesis. In insects, two gene products, TRH and TPH, are capable of converting tryptophan into 5-hydroxytryptophan (5-HTP). In the second step, 5-HTP is decarboxylated to serotonin by Dopa decarboxylase (DDC). Fig. S2. Effect of 5-HTP treatment on midgut and hindgut bacterial composition in B. dorsalis at genus level. Fig. S3. Effect of 5-HTP treatment on the relative abundance of S. marcescens and P. alcalifaciens in the gut of B. dorsalis. (a) Phylogenetic analysis of the identified S. marcescens and P. alcalifaciens operational taxonomic units (OTUs) with 18 related bacteria. The evolutionary history was inferred by using the neighbor-joining method conducted in MEGA 6 software. A sequence of Pseudomonas entomophila was used as the outgroup. Bootstrap values (percentages of 1,000 tree replications) greater than 50% are displayed at the nodes. Sequence GenBank accession numbers are shown in parentheses. (b, c) 5-HTP treatment increased the relative abundance of (b) S. marcescens and (c) P. alcalifaciens in the gut of B. dorsalis. Fig. S4. BdTPH regulates gut bacterial composition in B. dorsalis. (a) Histogram showing changes at the genus level. (b) Relative abundance of bacteria at the genus level in B. dorsalis gut postinjection of dsBdTPH and dsGFP by 16S rDNA sequencing. To restore the 5-HT level, BdTPH-silenced flies were treated with 1-mM 5-HTP by feeding after dsRNA injection. Fig. S5. BdTRH knockdown does not affect the gut commensal microbiome of B. dorsalis. (a) Gut BdTRH silencing efficiency in B. dorsalis at 72 h postinjection with 1.5 μg of dsGFP or dsBdTRH. (b) Effect of BdTRH silencing on gut total bacterial load. (c) Effect of BdTRH knockdown on gut S. marcescens burden. The housekeeping β-actin gene was used as an endo [file 12915_2022_1319_MOESM1_ESM.docx]

**
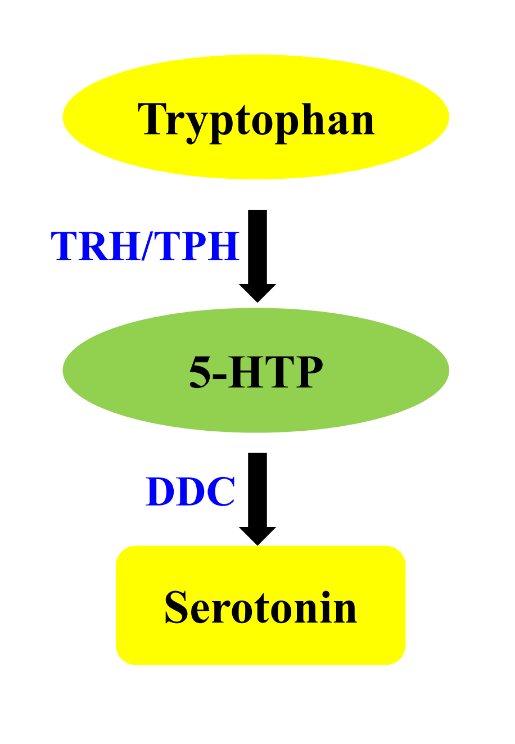
**

**Fig. S1** Schematic of 5-HT synthesis. Serotonin (5-HT) is synthesized in a two-step process. The amino acid tryptophan is first hydroxylated by Tryptophan hydroxylase (TPH). This is the rate limiting step in 5-HT synthesis. In insects, two gene products, TRH and TPH, are capable of converting tryptohan into 5-hydroxytryptophan (5-HTP). In the second step, 5-HTP is decarboxylated to serotonin by Dopa decarboxylase (DDC).


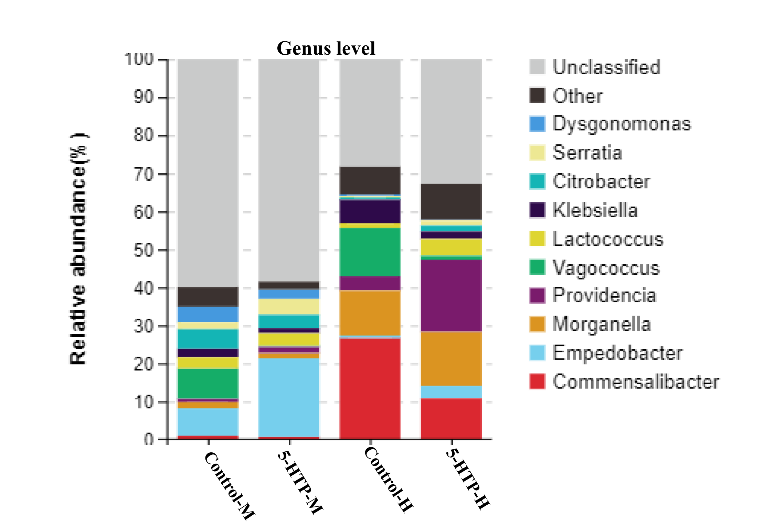


**Fig. S2** Effect of 5-HTP treatment on midgut and hidgut bacterial composition in *B. dorsalis* at genus level. M, midgut; H, hidgut.


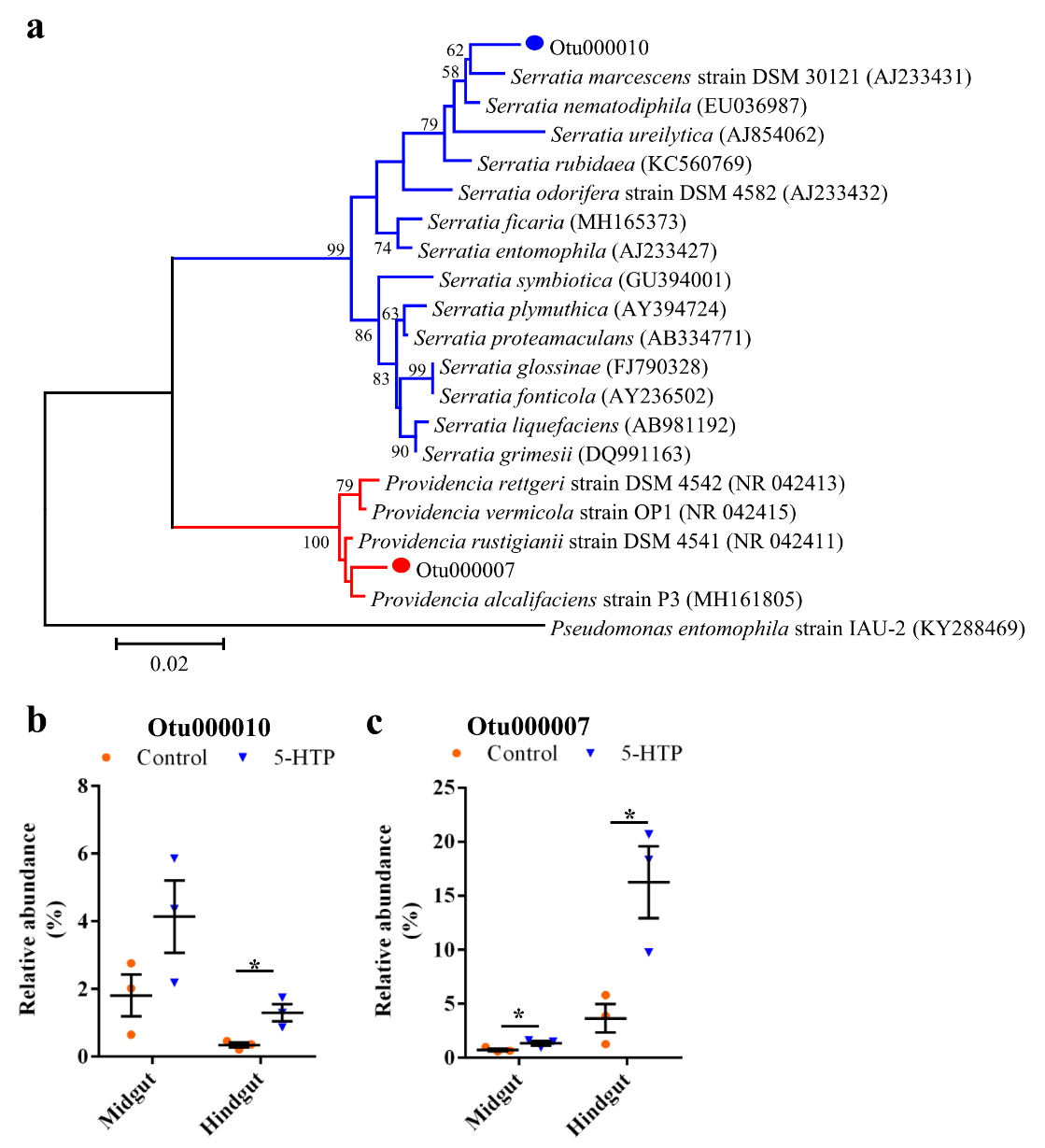


**Fig. S3** Effect of 5-HTP treatment on the relative abundance of *S. marcescens* and *P. alcalifaciens* in the gut of *B. dorsalis*. (**a**) Phylogenetic analysis of the identified *S. marcescens* and *P. alcalifaciens* operational taxonomic units (OTUs) with 18 related bacteria. The evolutionary history was inferred by using the neighbor-joining method conducted in MEGA 6 software. A sequence of *Pseudomonas entomophila* was used as the outgroup. Bootstrap values (percentages of 1,000 tree replications) greater than 50% are displayed at the nodes. Sequence GenBank accession numbers are shown in parentheses. (**b**, **c**) 5-HTP treatment increased the relative abundance of (**b**) *S. marcescens* and (**c**) *P. alcalifaciens* in the gut of *B. dorsalis*. Two-tailed unpaired t-test was performed for **b** and **c**. Error bars indicate ± s.e.m., *p<0.05, and three biological replicates were performed.


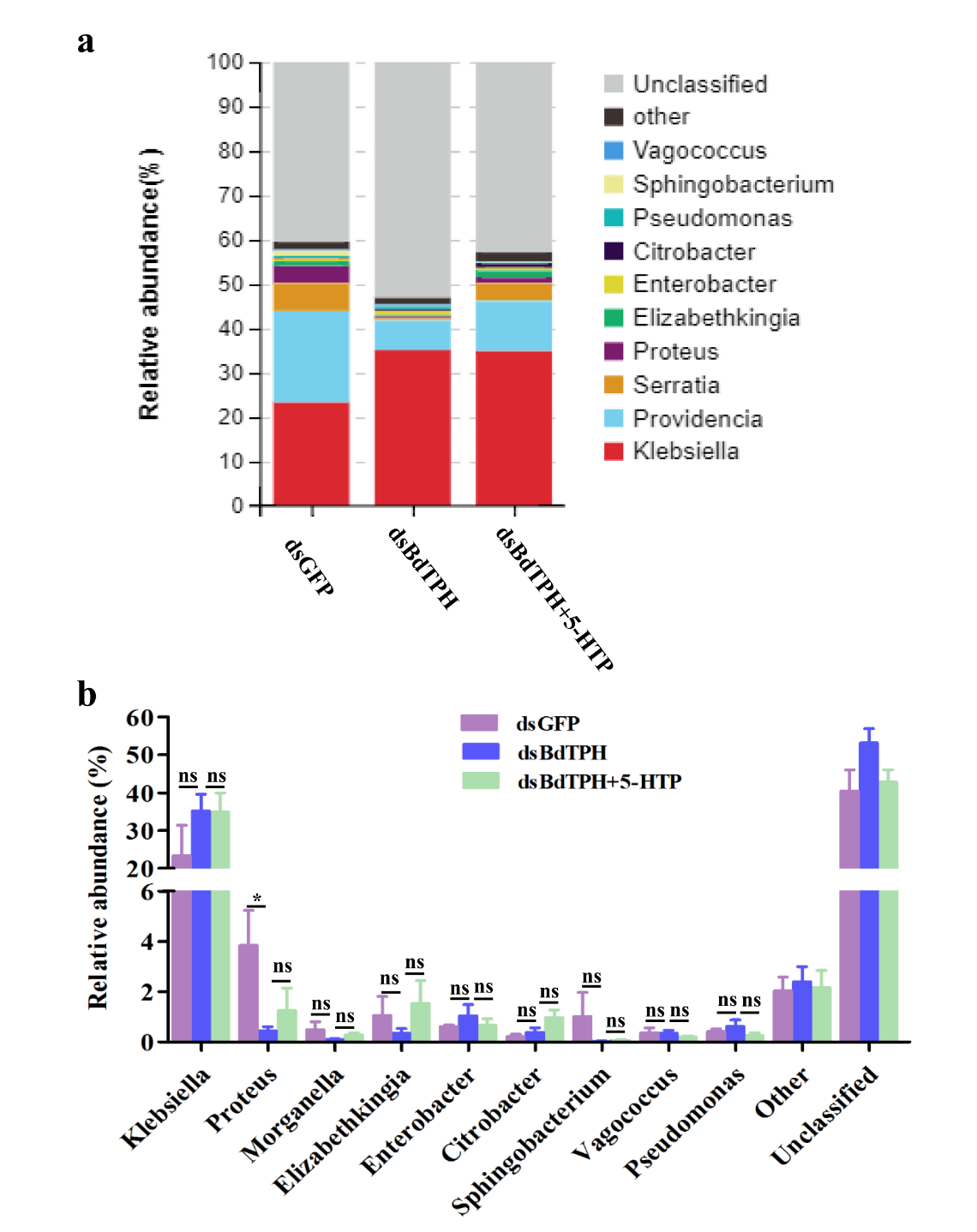


**Fig. S4** BdTPH regulates gut bacterial composition in *B. dorsalis*. (**a**) Histogram showing changes at the genus level. (**b**) Relative abundance of bacteria at the genus level in *B. dorsalis* gut postinjection of *dsBdTPH* and *dsGFP* by 16S rDNA sequencing. To restore the 5-HT level, *BdTPH*-silenced flies were treated with 1-mM 5-HTP by feeding after dsRNA injection. Data were analysed using student’s t-test. Error bars indicate ± s.e.m.; *p < 0.05, and ns means no significant difference.


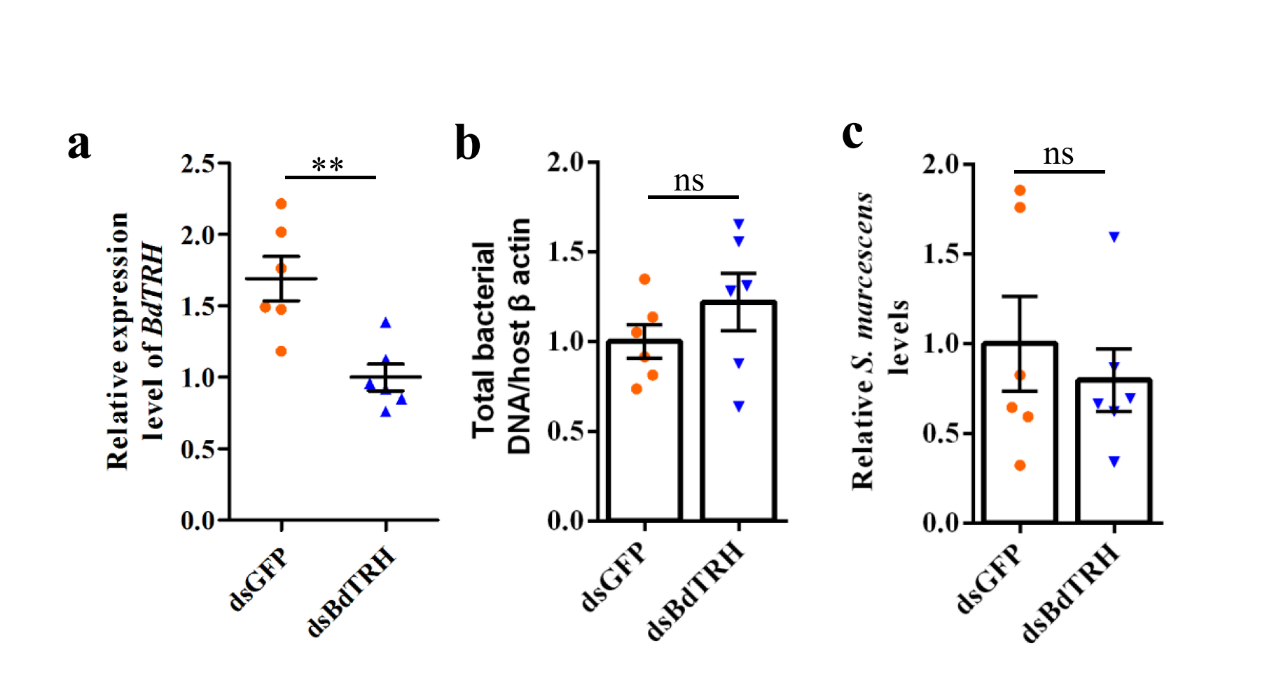


**Fig. S5** *BdTRH* knockdown does not affect the gut commensal microbiome of *B. dorsalis*. (**a**) Gut *BdTRH* silencing efficiency in *B. dorsalis* at 72 h postinjection with 1.5 µg of ds*GFP* or ds*BdTRH*. (**b**) Effect of *BdTRH* silencing on gut total bacterial load. (**c**) Effect of *BdTRH* knockdown on gut *S. marcescens* burden. The housekeeping β-actin gene was used as an endogenous control. Two-tailed unpaired t-test was performed in **a**, **b**, and **c**. Error bars indicate ± s.e.m.; *p < 0.05, **p < 0.01, ***p < 0.001, and ns means no significant difference. All results represent at least two independent experiments.


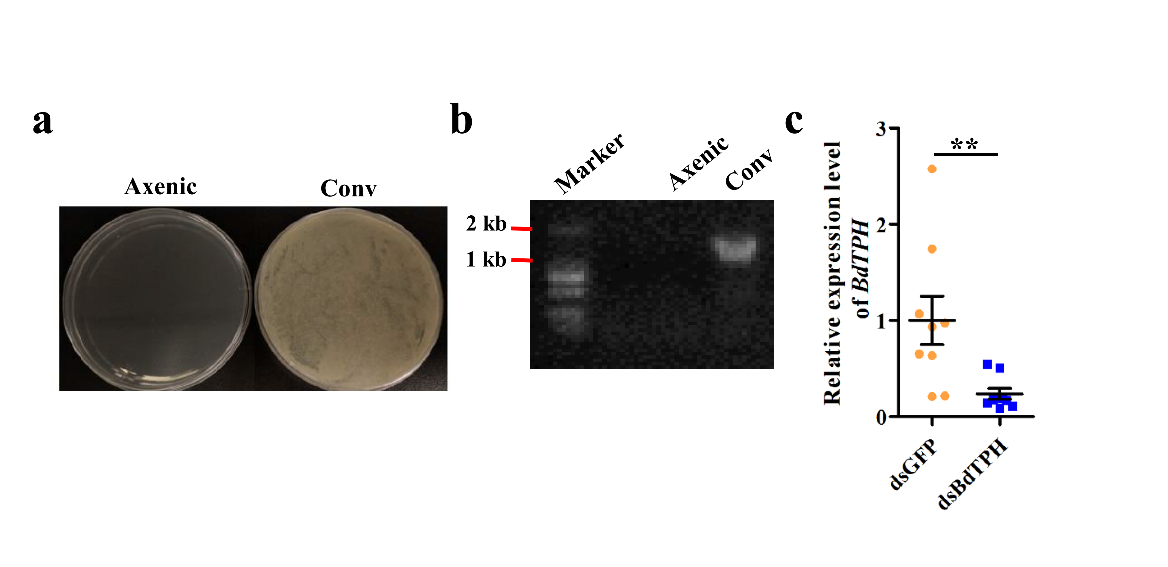


**Fig. S6** Gut *BdTPH* silencing efficiency in axenic *B. dorsalis*. (**a**-**b**) The efficacy of elimination of gut bacteria confirmed by culturing *B. dorsalis* gut homogenates on LB agar plates (**a**) and by performing PCR analysis (**b**) on gut homogenates using universal 16S rRNA gene primers. (**c**) The mRNA level of *BdTPH* in axenic *B. dorsalis* gut at 96 h postinjection with 2.0 µg of ds*GFP* or ds*BdTPH*. Data were normalized to expression levels in ds*GFP*-treated flies. Data were analysed using two-tailed unpaired t-test. Error bars indicate ± s.e.m.; **p < 0.01. Results represent at least two independent experiments.


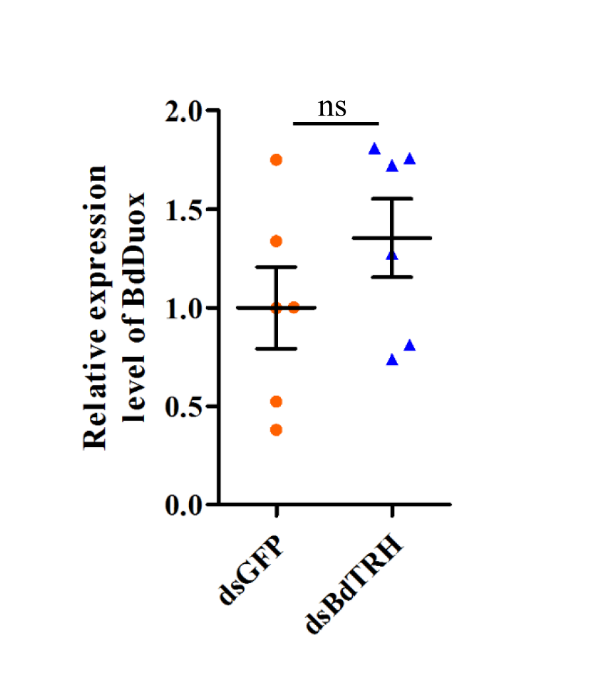


**Fig. S7** Effect of *BdTRH* silencing on gut *BdDuox* expression level in *B. dorsalis*. Two-tailed unpaired t-test was performed, and ns means no significant difference. Data are presented as mean ± s.e.m. All results were repeated in at least two independent experiments.


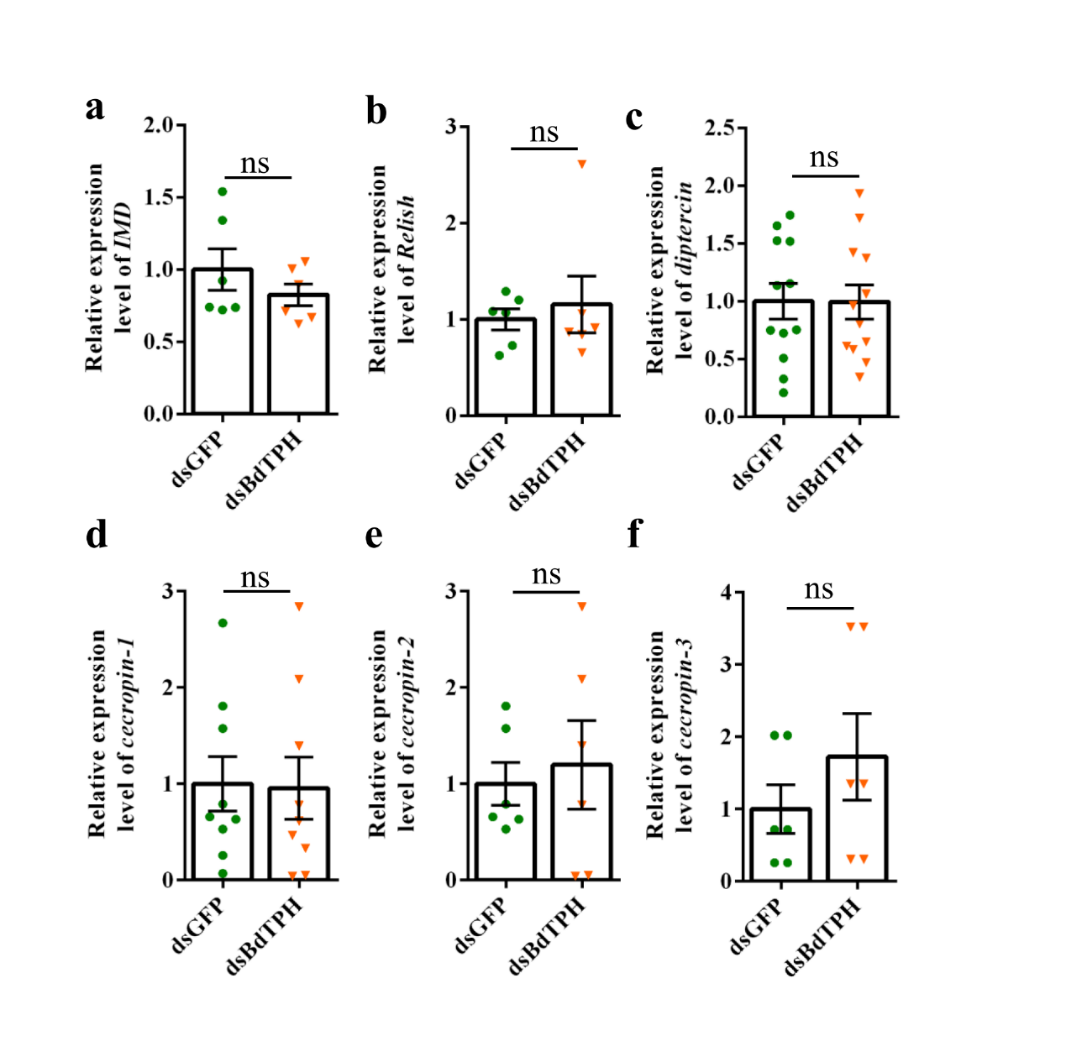


**Fig. S8** Expression of six genes related to IMD pathway in the guts of *BdTPH*-silencing *B. dorsalis*. The *GFP* dsRNA-treated flies served as controls. Gene expression of each sample was normalized to that of controls (taken as 1). Three biological replicates were conducted. Two-tailed unpaired t-test was performed for **a**-**f**. Error bars indicate ± s.e.m., ns means no significant difference. All results were repeated in at least two independent experiment.


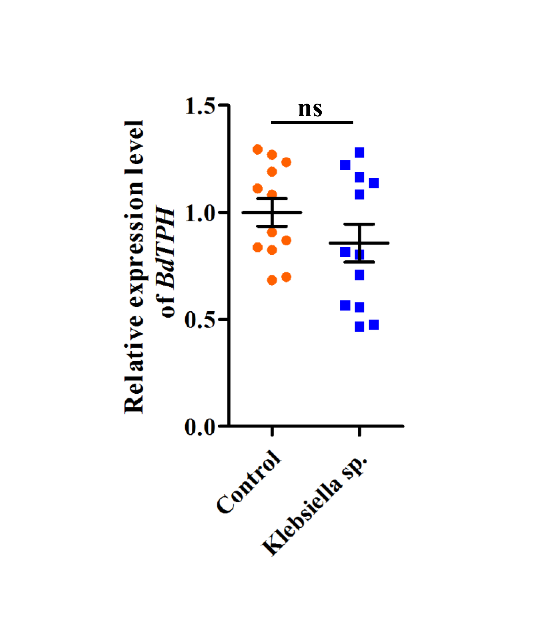


**Fig. S9** Relative expression levels of *BdTPH* after the bacteria *Klebsiella sp.* were introduced into the guts of antibiotic-treated flies. Two-tailed unpaired t-test was performed. Error bars indicate ± s.e.m., ns means no significant difference. All results were repeated in two independent experiments, with three to five biological replicates.


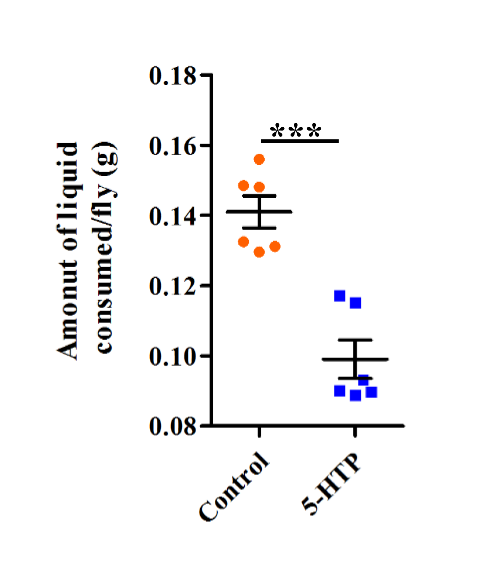


**Fig. S10** The effect of 1mM 5-HTP on liquid food consumption by *B. dorsalis*. Data were analysed using two-tailed unpaired t-test. Error bars indicate ± s.e.m.; ***p < 0.001. Methods: Two centrifuge tubes (5 ml), one containing 5% sucrose solution and another containing 1mM 5-HTP dissolved in 5% sucrose solution, were introduced into the plastic cages. The opening of the tube was plugged with cotton so that the solution did not flow out when inverted, but the flies could suck the solution from the tube. Tubes were labeled and weighed. Centrifuge tubes were removed after 96 h and weighed again to calculate the actual amount of food consumption. A separate control treatment without flies was run in parallel to observe the solvent water by evaporation. The control group was composed of the same material. The amount of food consumed by flies was calculated. Five-day-old *B. dorsalis* adults, a mix of the same number of males and females were used for the experiment. Three replicates were performed for each treatment. Each replicate contained 20 flies. Results were repeated in two independent experiments.
