# Supplementary material for: Serotonin modulates insect gut bacterial community homeostasis
Source: BMC Biol. 2022 May 13;20:105. doi: 10.1186/s12915-022-01319-x (PMC9103294; doi:10.1186/s12915-022-01319-x)
Supplement: Supplementary file 2 — Additional file 2: Table S1. Primers used in this study. [file 12915_2022_1319_MOESM2_ESM.docx]

**Table S1. Primers used in this study.**

| **The primers for genes cloning** | **Upper primer (5’-3’)** | **Lower primer (5’-3’)** |
| --- | --- | --- |
| BdTPH-ORF-complete | ATGATGGCCGAACGTCAGACGTC | CTACACCTTCAACTTCTCCACGGC |
| BdTRH- ORF-complete | AATGAGCGCTTCTGGTAAAAGTTTG | TTCCAAGGTCCAGCAGTTTCG |
| BdDuox | GTCGTTTATTCATGGGCGGC | GCGAGGACATACCCAGTAGC |
| AaTPH-ORF-complete | TGTTACTAAGGAGGTGTTTGTTGTGG | TTCAATCGTTTGGCACAGTCTTA |
| **The primers for Real-time PCR** | **Upper primer (5’-3’)** | **Lower primer (5’-3’)** |
| BdTPH-qPCR | GCAAGCCGATGTATACACCG | CCAGAAGATAGTCGCCAGCT |
| BdTRH-qPCR | GTATACGGCGCTGGACTACT | AGGGGCGTTGTATGCTATCA |
| BdDuox-qPCR | GACCACCACGTTTCTGGATG | TAACATCGGAAGCAGCAGA |
| BdNox-qPCR | ACCTGTCCGCGTTGTCATTT | AATGAGCGCTGATCACGGTT |
| BdIMD-qPCR | GAACCCATTTCGCCTAGTGG | ACATGTTTCCAACCTTCGCC |
| BdRelish-qPCR | TTGCAACATGATCACCGAGC | TAGCTAAGTCGCCGGCATTA |
| BdDiptericin-qPCR | CCCAAAGACAGCCTCAGTTCA | TTGCGAATACTGTCCGGTGG |
| BdCecropin1-qPCR | CATCTTCTTGGCTGTGGTGT | GACATTGGCGGCCTGTTG |
| BdCecropin2-qPCR | ATCTTCTTGGCCGTGGTGAT | CACGCTCCTGTCGAAAAGTA |
| BdCecropin3-qPCR | ACGACAAAACTAGTAAATCCCACCC | CTAATTTAGGCAGTGGCGGTTTGA |
| Bd-α-Tubulin-qPCR | CGCATTCATGGTTGATAACG | GGGCACCAAGTTAGTCTGGA |
| Bd-RpL32-qPCR | CCCGTCATATGCTGCCAACT | GCGCGCTCAACAATTTCCTT |
| Bd-β-actin-qPCR | TCGATCATGAAGTGCGATGT | ATCAGCAATACCGGGGTACA |
| AaTPH-qPCR | AAATCGGATTGGCTTCGCTC | GGTACTTTTGTTCGCCCGTT |
| AaDuox-qPCR | ATGCTGAGCCCAGAGAGATT | TTTCCTCATCAGTCCAATCG |
| AaS7-qPCR | GGGACAAATCGGCCAGGCTATC | TCGTGGACGCTTCTGCTTGTTG |
| AaActin-qPCR | GAACACCCAGTCCTGCTGACA | TGCGTCATCTTCTCACGGTTAG |
| *S. marcescens*-specific | TGCCTGGAAAGCGGCGATGG | CGCCAGCTCGTCGTTGTGGT |
| *P. alcalifaciens*-specific | TCTGCACGGTGTGGGTGTT | ACCGTCACGGCGGATTACT |
| Bacteria Universal 16s rDNA | ACTCCTACGGGAGGCAGCAG | ATTACCGCGGCTGCTGG |
| **The primers for double-strand RNA synthesis** | **Upper primer (5’-3’)** | **Lower primer (5’-3’)** |
| BdTPH-RNAi | ***TAATACGACTCACTATAGGG***ATGATG  GCCGAACGTCAGAC | ***TAATACGACTCACTATAGGG***CCATG  TTTATAGTTGTAAGC |
| BdTRH-RNAi | ***TAATACGACTCACTATAGGG***ATGAGC  GCTTCTGGTAAAAG | ***TAATACGACTCACTATAGGG***TATTT  TGCGTGGAAACCAGA |
| BdDuox-RNAi | ***TAATACGACTCACTATAGGG***ATCGTC  CGTCTACTCGTCGC | ***TAATACGACTCACTATAGGG***TAAGC  ATCTTCATCACATTC |
| AaTPH- RNAi | ***TAATACGACTCACTATAGGG***CGAATC  TGCCGAAGCGAAGA | ***TAATACGACTCACTATAGGG***CAGGT  TCTGGAAGACCACCC |
